# Supplementary material for: Interplay of spin–orbit coupling and Coulomb interaction in ZnO-based electron system
Source: Nat Commun. 2021 May 26;12:3180. doi: 10.1038/s41467-021-23483-4 (PMC8155003; doi:10.1038/s41467-021-23483-4)
Supplement: Supplementary file 1 — Supplementary Information [file 41467_2021_23483_MOESM1_ESM.pdf]

Supplementary Information: Interplay of spin-orbit coupling and Coulomb interaction in ZnO-based electron system

D. Maryenko,[1,*] M. Kawamura,[1] A. Ernst,[2,3] V. K. Dugaev,[4] E. Ya. Sherman,[5,6]
M. Kriener,[1] M. S. Bahramy,[7,†] Y. Kozuka,[8,9] and M. Kawasaki[1,7]

[1]*RIKEN Center for Emergent Matter Science (CEMS), Wako 351-0198, Japan*
[2]*Institute for Theoretical Physics, Johannes Kepler University, 4040 Linz, Austria*
[3]*Max Planck Institute of Microstructure Physics, D-06120 Halle, Germany*
[4]*Department of Physics and Medical Engineering, Rzeszów University of Technology, 35-959 Rzeszów, Poland*
[5]*Department of Physical Chemistry, University of the Basque Country UPV/EHU, Apartado 644, Bilbao 48080, Spain*
[6]*Ikerbasque, Basque Foundation for Science, Bilbao, Spain*
[7]*Department of Applied Physics and Quantum-Phase Electronics Center (QPEC), The University of Tokyo, Tokyo 113-8656, Japan*
[8]*Research Center for Magnetic and Spintronic Materials, National Institute for Materials Science (NIMS), Tsukuba 305-0047, Japan*
[9]*JST, PRESTO, Kawaguchi, Saitama 332-0012, Japan*

Supplementary Note 1: Evaluation of spin-orbit coupling coefficient α from experimental data

This section describes the derivation of Eq. (2) in the main text and discuss the possible consequences for the case of renormalized mass and SOC coefficient. The derivation relies on the fact that m and α are k wavevector independent and are the same for both branches of energy dispersion.

We consider a free electron system with the spin-orbit coupling, whose Hamiltonian is given by:

$$H_{\text{total}} = H_0 + H_{\text{SOC}} = \frac{\hbar^2 k^2}{2m} + \alpha(\sigma_x k_y - \sigma_y k_x), \tag{1}$$

where m is the electron effective mass and α is the spin-orbit coupling coefficient. The Hamiltonian has two eigenvalues

$$\varepsilon_{\pm} = \frac{\hbar^2 k^2}{2m} \pm \alpha k, \tag{2}$$

each describing the energy dispersion of the corresponding band, which cross at $k = 0$. We call the band described by ε_+ (ε_-) as the inner(outer) band.

Consider the realization where same for both bands Fermi energy ε_F lies above the crossing point at $k = 0$. Then the Fermi wavevectors for the inner and outer bands are:

$$k_{F,+} = \frac{m}{\hbar^2}\left[-\alpha + \sqrt{2\pi N \frac{\hbar^4}{m^2} - \alpha^2}\right] \tag{3}$$

$$k_{F,-} = \frac{m}{\hbar^2}\left[\alpha + \sqrt{2\pi N \frac{\hbar^4}{m^2} - \alpha^2}\right], \tag{4}$$

where N is the total electron density. At $\alpha = 0$, we introduce single $k_F \equiv k_{F,+} = k_{F,-}$, and the Fermi surface area for each band $A_F \equiv \pi k_F^2$ is associated with the oscillation frequency f of Shubnikov-de Haas oscillations by the relation:

$$f = \frac{\hbar}{2\pi e} A_F. \tag{5}$$

Now, in the experiment at $\alpha \neq 0$ (see Fig. 2 in the main text) we obtain two frequencies f_1 and f_2, which are associated with the inner and outer Fermi surfaces respectively. The area difference of two Fermi surfaces is:

$$\Delta A_F = \pi k_{F,-}^2 - \pi k_{F,+}^2 = 4\alpha \frac{\pi m}{\hbar^2}\sqrt{2\pi N - \frac{m^2}{\hbar^4}\alpha^2} \tag{6}$$

This relation establishes the connection between the strength of spin-orbit coupling α and the difference of Fermi pocket cross sections. On the other side

$$\Delta A_F = \frac{2\pi e}{\hbar}\Delta f = 4\pi^2 \Delta n. \tag{7}$$

where the notation $\Delta n \equiv e\Delta f/2\pi\hbar$ is introduced.

Then Supplementary Eqs. (6) and (7) yield:

$$\Delta n = \frac{\alpha m}{\pi\hbar^2}\sqrt{2\pi N - \frac{m^2}{\hbar^4}\alpha^2}. \tag{8}$$

Solving this equation one obtains:

$$\alpha^2 = \frac{\hbar^4\pi}{m^2}\left[N \pm \sqrt{N^2-(\Delta n)^2}\right]. \tag{9}$$

Since both solutions satisfy the request $\alpha^2 > 0$ and only one α can be the solution, we consider another restriction. If $\alpha = 0$, there should be only one oscillation frequency and thus $\Delta n = 0$. Then only one solution remains.

$$\alpha^2 = \frac{\hbar^4\pi}{m^2}\left[N - \sqrt{N^2-(\Delta n)^2}\right] = \frac{\hbar^4\pi}{m^2}\frac{N^2-(N^2-(\Delta n)^2)}{N+\sqrt{N^2-(\Delta n)^2}}. \tag{10}$$

It follows:

$$\alpha = \frac{\hbar^2}{m}\Delta n\frac{\sqrt{\pi}}{\sqrt{N+\sqrt{N^2-(\Delta n)^2}}}. \tag{11}$$

At $N \gg \left(m\alpha^2/\hbar^2\right)^2$ we have $\varepsilon_F \approx \pi\hbar^2 N/m$ and $\Delta n \ll N$. Thus, we obtain Eq. (2) of the main text:

$$\alpha = \frac{\hbar^2}{m}\Delta n\sqrt{\frac{\pi}{2N}}. \tag{12}$$

Supplementary Note 2: Estimation of electron effective mass

We demonstrate the estimation of the electron effective mass m from the Shubnikov-de Haas oscillations on the example of one $Mg_xZn_{1-x}O/ZnO$ structure, which is shown in the middle panel of Fig. 2a in the main text. We consider that the oscillation of magnetoresistance follows Lifshitz-Kosevich formalism [1]. We also consider only the leading term in Taylor series of Lifshitz-Kosevich approach:

$$\frac{\Delta R_{xx}}{R_{xx}} = \frac{4XT}{\sinh(XT)}\exp(-\pi/\omega_c\tau) \tag{13}$$
$$X = \frac{2\pi^2 k_B}{\hbar\omega_c},$$

where T is the temperature, $\omega_c = eB/m$ is the cyclotron frequency at field B, and k_B is the Boltzmann constant.

Supplementary Fig. 1a shows the development of oscillating part of the magnetoresistance with temperature, while panel (b) demonstrates the mass analysis according to Supplementary Eq. (13). The mass is estimated for several field values and is plotted in Supplementary Fig. 1c. At $N = 3.1 \times 10^{11}$ cm^{-2}, it amounts to m=0.41m_0, where m_0 is the free electron mass, enhanced compared to the bulk value of 0.32m_0. This enhancement is attributed to the correlation effects.

From the mass enhancement presented in Fig. 2c of the main text we can evaluate the corresponding parameter F_1^s, introduced in the Fermi liquid theory [2]. We use the relation $m^*/m = 1 + F_1^s/3$ and summarize the results in Supplementary Table I.

Supplementary Note 3: Renormalization of the effective mass and spin-orbit coupling by electron-electron interactions

Here we present a model for renormalization of the electron mass and spin-orbit coupling and their changes with the charge carrier density using the analysis based on the perturbation theory. The Hamiltonian of two-dimensional (2D) electron system with the Rashba SO coupling and electron-electron interaction is written as the sum of two terms

$$H = H_0 + H_{\text{int}}, \tag{14}$$

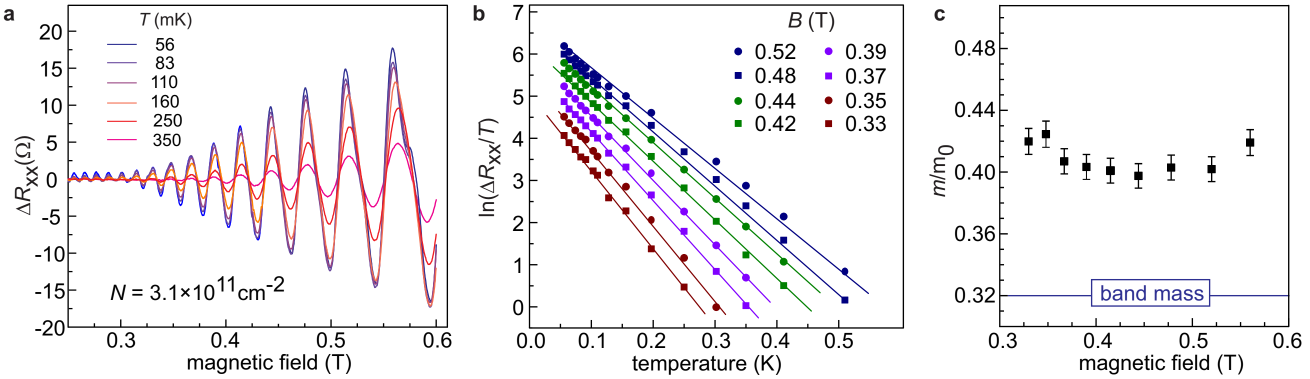

Supplementary Figure 1. **Estimate of electron mass.** **a** Oscillatory part of the magnetoresistance at various temperatures **b** Mass analysis according to Lifshitz-Kosevich approximation. The slope at a given magnetic field defines the electron effective mass. **c** Mass as a function of magnetic field. The mass enhancement is attributed to the electron correlation effects.The vertical error bars represent the standard deviation of the fit.

Supplementary Table I. Fermi liquid parameter F_1^s for ZnO structures. Here m_0 is the free electron mass

charge carrier density $[10^{11}\ \mathrm{cm}^{-2}]$	mass enhancement $m^*/0.32m_0$	F_1^s
1.3	1.88	2.63
2.04	1.65	1.96
3.1	1.31	0.93
6.1	1	0

where

$$H_0 = \psi^\dagger(\mathbf{r})\Big[-\frac{\hbar^2\Delta}{2m_b} - i\alpha_b\,\hat{\mathbf{z}}\cdot(\boldsymbol{\sigma}\times\boldsymbol{\nabla})\Big]\,\psi(\mathbf{r}), \qquad H_{\mathrm{int}} = v(\mathbf{r}-\mathbf{r}')\,\big[\psi^\dagger(\mathbf{r})\,\psi(\mathbf{r})\big]\big[\psi^\dagger(\mathbf{r}')\,\psi(\mathbf{r}')\big]. \tag{15}$$

Here m_b and α_b is the bare (interaction-independent) electron effective mass and the Rashba coupling constant, respectively, $\hat{\mathbf{z}}$ is the unit vector perpendicular to the x-y plane, $\psi^\dagger(\mathbf{r})$ and $\psi(\mathbf{r})$ are the spinor field operators, and the function $v(\mathbf{r})$ with $\mathbf{r} = (x, y)$ describes Coulomb interaction of electrons at a distance r. We assume that Coulomb interaction is screened, and its Fourier component is [3]

$$v(q) = \frac{2\pi e^2}{\epsilon\,(q+\kappa)}, \tag{16}$$

where ϵ is the dielectric constant and κ is the inverse screening length. The Schrödinger equation with Hamiltonian H_0 gives the two-band dispersion $\varepsilon_\lambda(k) = \varepsilon(k) \pm \alpha_b k$, where $\varepsilon(k) = \hbar^2k^2/2m_b$ and $\lambda = +(-)$ is the chirality index corresponding to the spin-related branch of the spectrum (cf. Supplementary Eq. (2)).

The exchange and Hartree diagrams [4] for the Coulomb interaction yield the following contributions to the self energy of electrons:

$$\hat{\Sigma}_{\mathrm{xc}}(\mathbf{k}) = i\int\frac{d\varepsilon}{2\pi}\frac{d^2k'}{(2\pi)^2}\,v(\mathbf{k}-\mathbf{k}')\,\hat{G}_0(\varepsilon,\mathbf{k}'), \tag{17}$$

$$\hat{\Sigma}_{\mathrm{H}}(\mathbf{k}) = -iv(0)\,\mathrm{tr}\int\frac{d\varepsilon}{2\pi}\frac{d^2k'}{(2\pi)^2}\,\hat{G}_0(\varepsilon,\mathbf{k}'), \tag{18}$$

where $\hat{G}_0(\varepsilon,\mathbf{k})$ is the 2×2 matrix Green's function of free electron corresponding to Hamiltonian H_0. After integrating the Green's function over ε we obtain

$$\int\frac{d\varepsilon}{2\pi}\,\hat{G}_0(\mathbf{k}',\varepsilon) = \frac{i}{2}\Big\{\theta[\mu-\varepsilon_+(k')] + \theta[\mu-\varepsilon_-(k')]\Big\}\,(1+\hat{\mathbf{z}}\cdot(\boldsymbol{\sigma}\times\mathbf{n}_{\mathbf{k}'})) \tag{19}$$

where μ is the chemical potential (at zero-temperature equivalent to the Fermi energy), $\hat{\mathbf{n}}_{\mathbf{k}}$ is the unit vector along $\mathbf{k}$ and $\theta(x)$ is the Heaviside step function. Substituting Supplementary Eqs. (16) and (19) into Supplementary Eqs. (17)

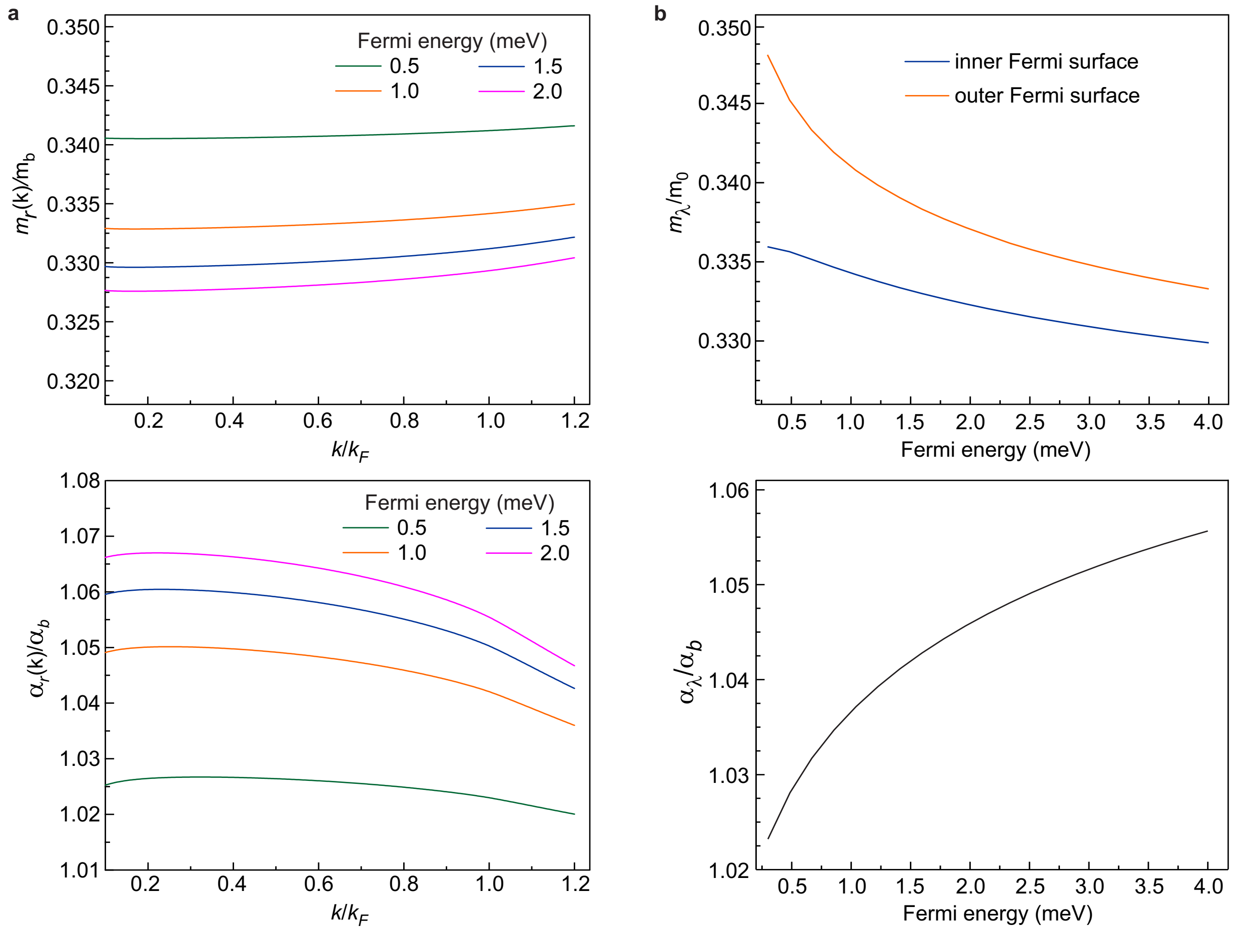

Supplementary Figure 2. **a** Renormalized by electron-electron interaction parameters $m_r(k) = m_b\chi_1^{-1}(k)$ and $\alpha_r(k) = \alpha_b\chi_2(k)$ in the self-consistent approach of Hamiltonian Supplementary Eg. (15) as a function of k/k_F for different chemical potentials μ. Here m_0 is the mass of free electron. **b** Effective mass m_λ (calculated with Supplementary Eq. (26)) and Rashba coupling α_λ at the Fermi surfaces, $k = k_{F,+}, k_{F,-}$, as a function of μ. The chirality index $\lambda = (+)$ describes the inner Fermi surface, while $\lambda = (-)$ describes the outer Fermi surface. Renormalized SOC coefficient α_r dependence on μ is the same for both Fermi surfaces.

and (18) we find

$$\hat{\Sigma}_{\rm xc}(\mathbf{k}) = -\frac{e^2}{2\pi\epsilon}\int_0^\pi d\varphi\int_0^{k_{F,+}}\frac{k'dk'}{\zeta(\mathbf{k},\mathbf{k}')+\kappa} - \frac{e^2\hat{\mathbf{z}}\cdot(\boldsymbol{\sigma}\times\mathbf{n_k})}{2\pi\epsilon}\int_0^\pi\cos\varphi d\varphi\int_0^{k_{F+}}\frac{k'dk'}{\zeta(\mathbf{k},\mathbf{k}')+\kappa}$$
$$-\frac{e^2}{2\pi\epsilon}\int_0^\pi d\varphi\int_0^{k_{F-}}\frac{k'dk'}{\zeta(\mathbf{k},\mathbf{k}')+\kappa} + \frac{e^2\hat{\mathbf{z}}\cdot(\boldsymbol{\sigma}\times\mathbf{n_k})}{2\pi\epsilon}\int_0^\pi\cos\varphi d\varphi\int_0^{k_{F-}}\frac{k'dk'}{\zeta(\mathbf{k},\mathbf{k}')+\kappa}, \tag{20}$$

$$\hat{\Sigma}_{\rm H}(\mathbf{k}) = \frac{e^2(k_{F+}^2+k_{F-}^2)}{2\epsilon\kappa}, \tag{21}$$

where the Fermi wavevectors $k_{F,\lambda}$ are determined by common chemical potential $\varepsilon_\lambda(k_{F,\lambda}) = \mu$ (cf. Supplementary Eqs. (3), (4), $\zeta(\mathbf{k},\mathbf{k}') = \sqrt{k^2+k'^2-2kk'\cos\varphi}$, and φ is the angle between vectors $\mathbf{k}$ and $\mathbf{k}'$. Note that Supplementary Eq. (20) contains the terms in form of spin-orbit interaction. The real part of self energy determines correction to the electron spectrum due to the interactions.

The Hartree contribution does not depend on k and, correspondingly, does not affect the energy structure. In our calculations we omit k-independent terms in Supplementary Eq. (21) assuming that they only lead to a uniform shift of all electron energies. The k-dependent contribution from the exchange diagrams can be written as

$$\hat{\Sigma}_{\rm xc}(\mathbf{k}) = \hat{\Sigma}^{(1)}_{\rm xc}(k) + \hat{\mathbf{z}}\cdot(\boldsymbol{\sigma}\times\mathbf{n_k})\,\hat{\Sigma}^{(2)}_{\rm xc}(k), \tag{22}$$

where the first and second terms stand for corresponding corrections to the Hamiltonian without interaction, $\hat{H}_{0\mathbf{k}} = \varepsilon(k) + \alpha_b \hat{\mathbf{z}} \cdot (\boldsymbol{\sigma} \times \mathbf{k})$. Note that in the limit $\alpha_b \to 0$, the first term, $\hat{\Sigma}^{(1)}_{\rm xc}(k)$ tends to a constant Supplementary Eq.(20) while $\hat{\Sigma}^{(2)}_{\rm xc}(k)$ vanishes since it is nonzero solely due to different Fermi momenta $\hbar k_{F,\lambda}$ in Supplementary Eq. (20).

These results are obtained in the first-order perturbation theory. Within this approach any higher order corrections to the spectrum should be small. To get more realistic results we use the self-consistent approach taking the Hamiltonian

$$H_{\mathbf{k}} = \varepsilon(k)\chi_1(k) + \alpha_b \, \hat{\mathbf{z}} \cdot (\boldsymbol{\sigma} \times \mathbf{k}) \, \chi_2(k), \tag{23}$$

where $\chi_{1,2}(k)$ are some unknown functions to be determined self-consistently. They can be presented as $\chi_1(k) = m_b/m_r(k)$ and $\chi_2(k) = \alpha_r(k)/\alpha$, where $m_r(k)$ and $\alpha_r(k)$ are the renormalized k-dependent parameters. Then in the first-order perturbation theory we get

$$\chi_1(k) \simeq 1 + \frac{\Sigma^{(1)}_{\rm xc}(k)}{\varepsilon(k)}; \qquad \chi_2(k) \simeq 1 + \frac{\Sigma^{(2)}_{\rm xc}(k)}{\alpha_b k}, \tag{24}$$

and in agreement with the perturbation approach, $\chi_1(k)$ and $\chi_2(k)$ are close to 1.

In frame of self-consistent approach we calculate the $\Sigma_{\rm xc}(\mathbf{k})-$dependent Green's function with the self energy calculated with full Green's function $\hat{G}(\mathbf{k}, \varepsilon)$, which takes into account interaction-induced nonparabolicity of the spectrum. Thus, in the previous equations we substitute $\varepsilon(k) \to \varepsilon(k)\chi_1(k)$ and $\alpha_b \to \alpha_b\chi_2(k)$. Correspondingly, we get the renormalized spectrum $\tilde{\varepsilon}_\lambda(k) = \varepsilon(k)\chi_1(k) \pm \alpha_b k \, \chi_2(k)$, where the Fermi wavevectors in each spin-related subband are determined as the solution of equation of common chemical potential μ as $\tilde{\varepsilon}_\lambda(k_{F,\lambda}) = \mu$.

We calculated the self energy and the spectrum by iterations starting with the 2D Fermi-gas realization $\chi_1(k) = \chi_2(k) = 1$, using the parameters: $m_b = 0.35\, m_0$, $\epsilon = 8.5$, and $\alpha_b = 2$ meVÅ. For the parameter κ we use an approximation, which takes into account the effect of Coulomb interaction on the screening. At $r_s \ll 1$ the value of κ can be determined by the random phase approximation (RPA) as $\kappa_0 = -(2\pi e^2/\epsilon)\, \Pi(\mathbf{0}, 0) = 2\pi e^2 \rho(\mu)/\epsilon$, where $\Pi(\mathbf{q}, \omega)$ is the polarization operator of two-dimensional electron gas and $\rho(\mu)$ is the density of states at Fermi level. Since the SO coupling is relatively weak, for $\mu \gg m_b \alpha_b^2/\hbar^2$ the chosen numerical parameters yield $\kappa_0 \simeq 1.27 \times 10^7$ cm^{-1}.

We assume that for $r_s \gg 1$ the value of κ is essentially renormalized by electron-electron interaction. For example, within the Landau theory for three-dimensional electron system one can get $\kappa \sim 1/r_s$ for $r_s \gg 1$. In the 2D case one can expect, based on the calculations of the interaction functions in the Landau theory for strongly screened potential (16), that at $r_s \gg 1$, the parameter κ tends to a r_s-independent constant. Thus, in our calculations we use an interpolation formula

$$\kappa = \frac{\kappa_0}{1 + \gamma r_s (1 + r_s)^{-1}}, \tag{25}$$

where γ is a constant and take $\gamma = 1$ as an example.

Supplementary Fig. 2a shows the behavior of functions $m_r(k) = m_b \chi_1^{-1}(k)$ and $\alpha_r(k) = \alpha_b \chi_2(k)$, which are k-dependent renormalized parameters of the mass and Rashba coupling. The function $k\alpha_r(k)$ determines the band splitting at each k. The main result presented in Supplementary Fig. 2a is that the electron-electron interaction-induced renormalization of $\alpha_r(k)$ and $m_r(k)$ makes them $k-$dependent and instead of bare constants α_b and m_b we obtain functions $\alpha_r(k)$ and $m_r(k)$. Correspondingly, the interactions modify the shape of dispersion curves to $\varepsilon_\lambda(k) = \hbar^2 k^2/2m_r(k) \pm \alpha_r(k)\, k$. Moreover, Supplementary Fig. 2a shows that all these functions depend on the density of electrons, which makes mass $m_r(k)$ smaller and $\alpha_r(k)$ larger with increasing electron density. As we see, $m_r(k)$ is lower than m_b and $\alpha_r(k)$ is larger than α_b for any k with this effect being stronger for larger μ. With increasing k the function $m_r(k)$ grows to the bare value m_b, whereas $\alpha_r(k)$ decreases to the bare α_b.

In addition, we find the renormalized electron cyclotron effective mass $m_\lambda(k)$ by using the standard definition

$$\frac{1}{m_\lambda(k)} = \frac{1}{\hbar^2 k} \frac{d\varepsilon_\lambda(k)}{dk}, \tag{26}$$

defined for each branch of the spectrum, $\varepsilon_\lambda(k)$.

Taking the effective mass $m_\lambda(k)$ and parameter $\alpha_r(k)$ at the Fermi surfaces ($k = k_{F,\lambda}$), corresponding to given value of μ, we obtain the dependence of redefined quantities $m_\lambda \equiv m_\lambda(k_{F,\lambda})$ and $\alpha_\lambda \equiv \alpha_r(k_{F,\lambda})$ on the chemical potential. These dependences are presented in Supplementary Fig. 2b, where m_λ decreases whereas α_λ increases with the increase in the chemical potential and, therefore, in the electron density.

Earlier, the effect of electron-electron interaction in spin-orbit coupling has been considered in Ref. [5]. It was found that in frame of the RPA the interaction reduces effective mass and enhances spin-orbit coupling. This is in agreement with our calculations (see Supplementary Fig. 2b, where $m_\lambda < m_b$ and $\alpha_\lambda > \alpha_b$ for all μ). However, as we see, the variation of the mass m_λ and α_λ with chemical potential μ corresponds to increase of m_λ and decrease of α_λ at smaller density of electrons.

Qualitatively, theoretical results are in some agreement with the experiment (see Supplementary Fig. 2b) but quantitatively the disagreement is rather strong. The main reason is that in strong-interaction regime the existing theory cannot describe the electron spectrum but allows to relate certain interaction parameters with observable quantities.

Supplementary Note 4: Defects at $Mg_xZn_{1-x}O/ZnO$ interfaces

Various defects such as oxygen vacancies, anti-site or interstitial defects can lead to a strong change of the effective band mass at the Fermi energy. To demonstrate this fact, we simulated these types of defects at the $Mg_{0.05}Zn_{0.95}O$ using a coherent potential approximation as it is implemented within the multiple scattering theory for semi-infinite systems [6]. The concentration of impurities was fixed in our simulations to be 0.1%. The results are presented in Supplementary Fig. 3, which show the spectral function corresponding to the conductance band in $Mg_{0.05}Zn_{0.95}O$ for three types of defects: (i) oxygen vacancies (Supplementary Fig. 3(a)); (ii) Zn-O anti-site defects (Supplementary Fig. 3(b)); (iii) oxygen interstitial defects (Supplementary Fig. 3(c)). All types of considered defects demonstrate a significant enhancement of the effective band mass in the vicinity of the Fermi energy. Our simulations should only mimic the impact of defects on the conductance band, however we have no information about the real defect structure at $Mg_xZn_{1-x}O/ZnO$ interfaces. In accordance with our previous studies, all these defect type are energetically possible in the ground state.

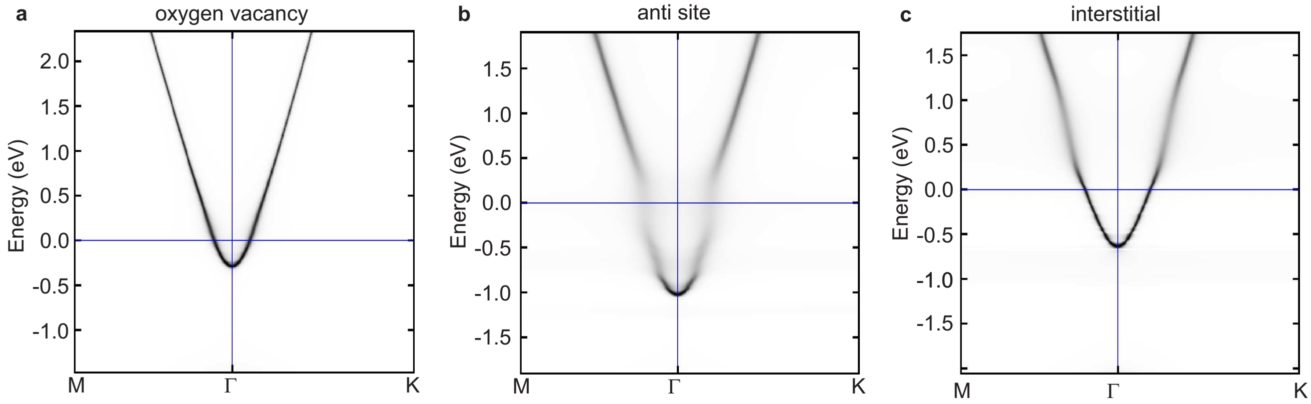

Supplementary Figure 3. Spectral function of the $Mg_{0.05}Zn_{0.95}O$ in presence of defects (0.1%): **a** oxygen vacancies; **b** Zn-O anti-site defects; **c** oxygen interstitial defects.

Supplementary Note 5: Mass renormalization due to piezoelectric electron-phonon coupling at the $Mg_xZn_{1-x}O/ZnO$ interface

Since ZnO is a strong piezoelectric where electron-phonon coupling modifies the electron effective mass measured in the cyclotron resonance experiments in bulk crystal [7], it would be of interest to see the effect of this coupling in two-dimensional electron systems at the $Mg_xZn_{1-x}O/ZnO$ interface. Here electrons interact with two kinds of acoustic phonons, the longitudinal (l) and the shear (s) ones. Taking into account that the speed of the lower-frequency shear sound in ZnO is $s_s = 2.1 \times 10^5$ cm/s, the typical phonon energy at the wave vector $q = 10^6$ cm^{-1}, corresponding to the Fermi momentum at electron concentration $\sim 10^{11}$ cm^{-2} is $\hbar s_s q \sim 0.1$ meV is too high for the phonon to be excited at experimental temperature 40 mK. For this reason, a quantum single-phonon perturbation theory is sufficient for calculation of the renormalized effective mass. Here we will use the approach proposed by Hutson [8] and Mahan and Hopfield [7] to evaluate the corresponding renormalization of the electron mass.

The electron-phonon coupling Hamiltonian in terms of electron ($a^\dagger_{\mathbf{k}+\mathbf{q}}, a_\mathbf{k}$) and phonon ($b^\dagger_{-\mathbf{q}}, b_\mathbf{q}$) creation and annihilation operators has the form:

$$H_{\mathrm{e-ph}} = \sqrt{\frac{1}{V}} \sum_{\nu,\mathbf{q}} \frac{\widetilde{e}\sqrt{\hbar}}{\sqrt{\rho s_\nu q}} f_\nu(\mathbf{q}) \left(b^\dagger_{-\mathbf{q}} + b_\mathbf{q} \right) a^\dagger_{\mathbf{k}+\mathbf{q}} a_\mathbf{k}, \tag{27}$$

where index ν denotes the phonon mode ($\nu = l$, longitudinal and $\nu = s$, shear) with velocity s_ν and wavevector $\mathbf{q}$, $f_\nu(\mathbf{q})$ includes the strength of piezocoupling, V is the crystal volume, ρ is the crystal density, and the effective charge $\widetilde{e} = e/\epsilon$. Here and below we neglect the change in the phonon properties at the $\mathrm{Mg}_x\mathrm{Zn}_{1-x}/\mathrm{ZnO}$ interface. Neglecting small phonon frequency [7], we use perturbation theory for the k-dependent energy shift $\delta\varepsilon_{\mathrm{e-ph}}(k)$:

$$\delta\varepsilon_{\mathrm{e-ph}}(k) = \sum_\nu \left\langle f^2_\nu(\mathbf{q}) \right\rangle \frac{\widetilde{e}^2}{\rho s_\nu} \frac{\hbar}{(2\pi)^3} \int_0^Q q dq \int_0^\pi \sin\theta d\theta \int_0^{2\pi} \frac{d\phi}{\varepsilon(\mathbf{k}) - \varepsilon(\mathbf{k}+\mathbf{q}_\|)}, \tag{28}$$

where Q is the cutoff wave vector due to the finite width of the electron wavefunction at the interface, and $\left\langle f^2_\nu(\mathbf{q}) \right\rangle$ stands for the averaging over directions of $\mathbf{q}$. The in-plane phonon wavevector is given by: $q_\| = q\sin\theta > 0$ (where θ is the corresponding polar angle), therefore

$$\varepsilon(\mathbf{k}) - \varepsilon(\mathbf{k}+\mathbf{q}_\|) = -\frac{\hbar^2}{2m_b}\left(q^2_\| + 2kq_\| \cos\phi \right), \tag{29}$$

where ϕ is the azimuthal angle.

First integrating over ϕ we obtain

$$\int_0^{2\pi} \frac{d\phi}{\varepsilon(\mathbf{k}) - \varepsilon(\mathbf{k}+\mathbf{q}_\|)} = -\frac{2m_b}{q_\|\hbar^2} \frac{2\pi}{\sqrt{q^2_\| - (2k)^2}} \tag{30}$$

and note that we need $q_\| > 2k$ to get a nonzero integral and, therefore, $q \geq 2k$. Further integration over the polar angle at $q \gg 2k$ yields:

$$\int_0^\pi \frac{d\theta}{\sqrt{\sin^2\theta - (2k/q)^2}} = \int_{\arcsin(2k/q)}^{\pi - \arcsin(2k/q)} \frac{d\theta}{\sqrt{\sin^2\theta - (2k/q)^2}} = (4\ln 2)\ln\frac{q}{2k}. \tag{31}$$

In the large $q \gg 2k$ limit we obtain further

$$\int_{2k}^Q \frac{dq}{q} \ln\frac{q}{2k} = \frac{1}{2}\ln^2\left(\frac{2k}{Q}\right). \tag{32}$$

It is convenient to introduce the Fermi velocity v_F and to write, as in Supplementary Eq. (26), the renormalized mass at the Fermi surface, m, as:

$$\frac{m_b}{m} = 1 + \frac{2\ln 2}{\pi^2} \sum_\nu \frac{\left\langle f^2_\nu(\mathbf{q}) \right\rangle}{\epsilon\rho s^2_\nu} \frac{e^2}{\epsilon\hbar v_F} \frac{s_\nu}{v_F} \ln\left(\frac{Q}{2k_F}\right), \tag{33}$$

where k_F is the Fermi wavevector, and $\ln(2k_F/Q) < 0$ by requirement of the narrow density distribution along the $z-$axis. Note that here $e^2/\epsilon\hbar$ is the excitonic electron velocity ($\sim 3 \times 10^7$ cm/s) with $e^2/\epsilon\hbar v_F \gg 1$.

Using the symmetry analysis of Hutson [8], we obtain that at $Q/2k_F \gg 1$, the main contribution to the mass renormalization is due to the longitudinal phonons ($\nu = l$) and the angular averaging $\langle f^2_l(\mathbf{q})\rangle$ can be easily performed in this case. For three-dimensional electrons the values $\langle f^2_l(\mathbf{q})\rangle/\epsilon\rho s^2_\nu$ are given by the electromechanical coefficient $\left(K^2_l\right)_{\mathrm{av}} = 0.012$. Taking into account that in 2DES electron mainly interact with the phonons propagating along the $z-$axis, we obtain $\left\langle f^2_l(\mathbf{q}) \right\rangle / \epsilon\rho s^2_l \approx 10 \left(K^2_l\right)_{\mathrm{av}}$. For the given material parameters $s_l = 5 \times 10^5$ cm/s and typical Fermi velocity $v_F = 3 \times 10^6$ cm/s we obtain as a result the difference $|m_b/m - 1|$ less than 0.1 and, more important, m increases with increasing the Fermi momentum. This is in agreement with the result of Mahan and Hopfield [7] and in contrast to the experimental observation presented in the main text.

Supplementary Note 6: Strain effect at the $Mg_xZn_{1-x}O$/ZnO interface

The Mg content in our structures varies from $\approx$ 1% for the lowest electron density sample to $\approx$ 5% for the highest electron density sample, respectively. For the largest $x \approx 0.05$, the out-of-plane lattice constant of $Mg_xZn_{1-x}O$ shrinks by 0.0035 Åcompared to the ZnO out-of-plane lattice constant c=5.204 Å[9]. In the crystal growth procedure the $Mg_xZn_{1-x}O$ and ZnO are coherently connected at the interface. As a result, strictly speaking, the entire structure experiences some strain at the interface. However, due to a very small lattice constants mismatch, the strain is weak and does not produce a visible effect. Furthermore, the electron system resides mostly in ZnO with only a small portion of the electron probability density penetrating into the $Mg_xZn_{1-x}O$ layer. Considering additionally that the strain of $Mg_xZn_{1-x}O$ does not reach much into the ZnO layer, the change in the spin-orbit coupling due to the strain effect is negligible.

Supplementary Note 7: Electron wave function width at the $Mg_xZn_{1-x}O$/ZnO interface

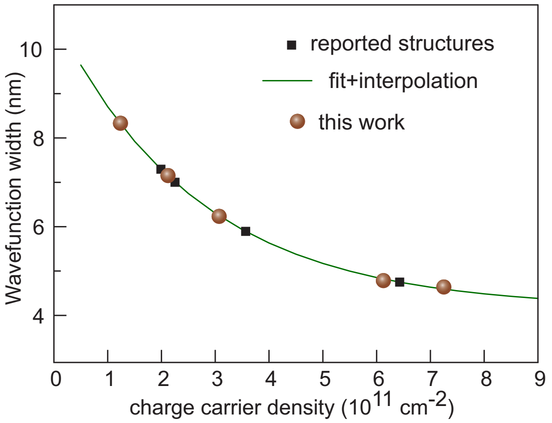

Supplementary Figure 4. **Width of wavefunction**.

The section describes the calculation of $\langle k_z^2 \rangle$ for analysing the spin-orbit coupling effect shown in Fig. 4b of the main text. Electron wavefunction at the interface with a triangular-like confinement potential can be presented in the Fang-Howard form as [3]:

$$\psi(z) = \frac{\xi^{3/2}}{\sqrt{2}} z e^{-\xi z/2}, \tag{34}$$

where ξ defines the extension perpendicular to the interface, with the corresponding expectation values:

$$\langle k_z^2 \rangle \equiv -\int_0^\infty \psi(z)\psi''(z)dz = \frac{\xi^2}{4}, \qquad \langle z^2 \rangle \equiv \int_0^\infty z^2\psi^2(z)dz = \frac{12}{\xi^2}. \tag{35}$$

The energy minimization yields the relation between $\langle k_z^2 \rangle$ and the electric field E at the interface

$$\langle k_z^2 \rangle = \frac{(12)^{2/3}}{4} \left(\frac{eEm}{\hbar^2} \right)^{2/3}, \tag{36}$$

where the field at the electron concentration N can be estimated using the electrical neutrality of the total interface as:

$$E = \frac{2\pi e N}{\epsilon}. \tag{37}$$

By using Supplementary Eqs. (36) and (37) and the definition of parameters in subsections 1 and 3, we obtain the ratio $k_F^2/\langle k_z^2 \rangle \approx 2/\left(3r_s\right)^{2/3}$. For strong correlations, where the Wigner-Seitz parameter $r_s \gg 1$, one has $k_F^2/\langle k_z^2 \rangle \ll 1$, and, therefore, $b\langle k_z^2 \rangle - k_\parallel^2$ in Eq. (1) of the main text is close to $b\langle k_z^2 \rangle$.

For the choice of the wavefunction shape in Supplementary Eq. (34) we have with a high accuracy

$$\langle z^2 \rangle^{1/2} \approx 0.7 d_{\mathrm{wf}}, \tag{38}$$

where d_{wf} is the full width of the $\psi(z)-$function at half-maximum. Therefore, $\langle k_z^2 \rangle \approx 6/d_{\mathrm{wf}}^2$. In our previous studies using optical probing of the interface we reported the values of d_{wf} [10]. Note that the above formulas yield at $N = 10^{12}$ cm^{-2} the width $\langle z^2 \rangle^{1/2} \approx 4.0$ nm, close to Ref. [10].

Filled black squares in Supplementary Fig. 4 represent FWHM d_{wf} as a function of electron density N. The phenomenological fit for N expressed in the units of 10^{11} cm^{-2}, $d_{\mathrm{wf}} = 4.16 + 6.62 \exp(-N/2.67)$ (green line) describes well the d_{wf} dependence on N. Filled circles represent the samples used in the current work. Since the heterostructure design of the current samples is the same as of those used in Ref. [10], the $N-$dependence of the wavefunction width is estimated using the same phenomenological dependence. Knowing the FWHM of the electron states in our samples we find the corresponding ξ-parameter, summarized in the Table II.

Supplementary Table II. Tabulated FWHM of the wavefunction and the corresponding ξ-parameter.

N [10^{11} cm^{-2}]	FWHM [nm]	ξ [nm^{-1}]
1.3	8.2	0.60
2.04	7.2	0.68
3.09	6.2	0.79
6.09	4.8	1.02
7.25	4.6	1.06

Supplementary Note 8: Table of various materials

Summary of electron system parameters for Fig.4c of the main text.

Supplementary Table III. Comparison of different semiconductors. $k_{\mathrm{SOC}} = m\alpha/\hbar^2$

material system	SOC coefficient [meV·Å]	n [10^{11} cm^{-2}]	m/m_0	k_{SOC} [μm^{-1}]
InSb/InAlSb [11]	130	3	0.014	2.4
InGaAs/InAlAs [12]	70	20	0.046	4.25
InAs/AlSb [13]	60	15	0.04	3.17
GaN/AlGaN [14]	6	10	0.22	1.74
GaAs (electrons) [15]	1-4	5	0.067	0.35
SiGe/Si/SiGe [16]	0.05	5	0.19	0.0125
InSe [17]	100	80	0.14	18.3

* maryenko@riken.jp
† Current address: Department of Physics and Astronomy, The University of Manchester, Oxford Road, Manchester M13 9PL, United Kingdom

[1] I. M. Lifshitz, and L. M. Kosevich, On the theory of the Shubnikov-de Haas effect, *Soviet Physics JETP* **6**, 67 (1958).
[2] E.M. Lifshitz and L. P. Pitaevskii, *Statistical Physics: Theory of the Condensed State (Course of Theoretical Physics Vol. 9)* (Butterworth-Heinemann) 1980.
[3] T. Ando, A. B. Fowler, and F. Stern, Electronic properties of two-dimensional systems, *Rev. Mod. Phys.* **54**, 437 (1982).
[4] A. A. Abrikosov, L. P. Gorkov, and I. E. Dzyaloshonski, *Methods of Quantum Field Theory in Statistical Physics* (Dover Books on Physics) 1975.

[5] G.-H. Chen and M. E. Raikh, Exchange-induced enhancement of spin-orbit coupling in two-dimensional electronic systems, *Phys. Rev. B* **60**, 4826 (1999).

[6] M. Hoffmann, A. Ernst, W. Hergert, V. N. Antonov, W. A. Adeagbo, R. M. Geilhufe, and H. Ben Hamed, Magnetic and Electronic Properties of Complex Oxides from First Principles, *Physica status solidi (b)* **257**, 1900671 (2020).

[7] G. D. Mahan and J. J. Hopfield, Piezoelectric Polaron Effects in CdS, *Phys. Rev. Lett.* **12**, 241 (1964).

[8] A. R. Hutson, Piezoelectric Scattering and Phonon Drag in ZnO and CdS, *J. Appl. Phys.* **32**, 2287 (1961).

[9] Y. Kozuka, A. Tsukazaki, and M. Kawasaki, Challenges and oportunities of ZnO-related single crystalline heterostructures, *Appl. Phys. Rev.* **1**, 011303 (2014).

[10] V. V. Solovyev, A. Van'kov, I. Kukushkin, J. Falson, D. Zhang, D. Maryenko, Y. Kozuka, A. Tsukazaki, J. H. Smet, and M. Kawasaki, Optical probing of MgZnO/ZnO heterointerface confinement potential energy levels, *Appl. Phys. Lett.* **106**, 082102 (2015).

[11] A. Gilbertson, W. R. Branford, M. Fearn, L. Buckle, P. Buckle, T. Ashley, and L. F. Cohen, Zero-field spin splitting and spin-dependent broadening in high-mobility InSb/$In_{1-x}Al_xSb$ asymmetric quantum well heterostructures, *Phys. Rev. B* **79**, 235333 (2009).

[12] J. Nitta, T. Akazaki, H. Takayanagi, and T. Enoki, Gate Control of Spin-Orbit Interaction in an Inverted $In_{0.53}Ga_{0.47}As/In_{0.52}Al_{0.48}As$ Heterostructure, *Phys. Rev. Lett.* **78**, 1335 (1997).

[13] J. P. Heida, B. J. van Wees, J. J. Kuipers, T. M. Klapwijk, and G. Borhgs, Spin-orbit interaction in a two-dimensional electron gas in a InAs/AlSb quantum well with gate-controlled electron density, *Phys. Rev. B* **57**, 11911 (1998).

[14] S. Schmult, M. J. Manfra, A. Punnoose, A. M. Sergent, K. W. Baldwin, and R. J. Molnar, Large Bychkov-Rashba spin-orbit coupling in high-mobility GaN/$Al_xGa_{1-x}N$ heterostructures, *Phys. Rev. B* **74**, 033302 (2006).

[15] M. P. Walser, C. Reichl, W. Wegscheider, and S. Salis, Direct mapping of the formation of a persistent spin helix, *Nature Physics* **8**, 757 (2012).

[16] Z. Wilamowski, W. Jantsch, H. Malissa, and U. Rössler, Evidence and evaluation of the Bychkov-Rashba effect in SiGe/Si/SiGe quantum wells, *Phys. Rev. B* **66**, 195315 (2002).

[17] Premasiri Premasiri, Santosh Kumar Radha, Sukrit Sucharitakul, U. Rajesh Kumar, Raman Sankar,, Fang-Cheng Chou, Yit-Tsong Chen, and Xuan P. A. Gao, Tuning Rashba Spin-Orbit Coupling in Gated Multilayer InSe, *NanoLetters* **18**, 4403 (2018).
